# Targeted Protein Degradation Phenotypic Studies Using HaloTag CRISPR/Cas9 Endogenous Tagging Coupled with HaloPROTAC3

**DOI:** 10.1002/cpph.81

**Published:** 2020-12-17

**Authors:** Elizabeth A. Caine, Sarah D. Mahan, Rebecca L. Johnson, Amanda N. Nieman, Ngan Lam, Curtis R. Warren, Kristin M. Riching, Marjeta Urh, Danette L. Daniels

**Affiliations:** ^1^ Promega Corporation Madison Wisconsin; ^2^ Boehringer Ingelheim Pharmaceuticals Ridgefield Connecticut

**Keywords:** CRISPR, HaloPROTAC3, HaloTag, HiBiT, phenotype, PROTAC, targeted protein degradation, VHL

## Abstract

To assess the role of a protein, protein loss phenotypic studies can be used, most commonly through mutagenesis RNAi or CRISPR knockout. Such studies have been critical for the understanding of protein function and the identification of putative therapeutic targets for numerous human disease states. However, these methodological approaches present challenges because they are not easily reversible, and if an essential gene is targeted, an associated loss of cell viability can potentially hinder further studies. Here we present a reversible and conditional live‐cell knockout strategy that is applicable to numerous proteins. This modular protein‐tagging approach regulates target loss at the protein, rather than the genomic, level through the use of HaloPROTAC3, which specifically degrades HaloTag fusion proteins via recruitment of the VHL E3 ligase component. To enable HaloTag‐mediated degradation of endogenous proteins, we provide protocols for HaloTag genomic insertion at the protein N or C terminus via CRISPR/Cas9 and use of HaloTag fluorescent ligands to enrich edited cells via Fluorescence‐Activated Cell Sorting (FACS). Using these approaches, endogenous HaloTag fusion proteins present in various subcellular locations can be degraded by HaloPROTAC3. As detecting the degradation of endogenous targets is challenging, the 11‐amino‐acid peptide tag HiBiT is added to the HaloTag fusion to allows the sensitive luminescence detection of HaloTag fusion levels without the use of antibodies. Lastly, we demonstrate, through comparison of HaloPROTAC3 degradation with that of another fusion tag PROTAC, dTAG‐13, that HaloPROTAC3 has a faster degradation rate and similar extent of degradation. © 2020 The Authors.

**Basic Protocol 1**: CRISPR/Cas9 insertion of HaloTag or HiBiT‐HaloTag

**Basic Protocol 2**: HaloPROTAC3 degradation of endogenous HaloTag fusions

## INTRODUCTION

**Figure 1:**
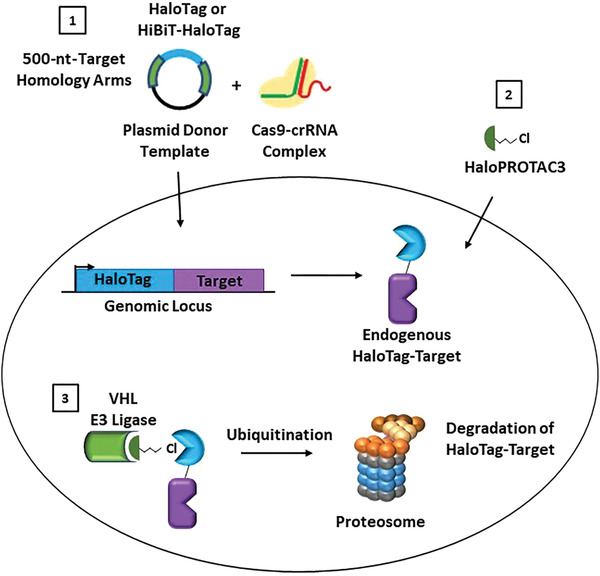
Schematic of HaloPROTAC3 degradation of HaloTag protein fusions in live cells. First, CRISPR/Cas9 technology is used to insert HaloTag or HiBiT‐HaloTag into the genomic locus of the target protein using a dsDNA donor plasmid and Cas9‐crRNA complex (1). After the HaloTag fusion is expressed, cells are treated with HaloPROTAC3(2). HaloPROTAC3 induces a ternary complex between the VHL E3 ligase component and HaloTag protein fusion, resulting degradation of the HaloTag target protein via the ubiquitin‐proteasomal pathway (3).

Targeted protein degradation using proteolysis targeting chimeras (PROTACs) is a rapidly growing research area and an exciting new modality of therapeutic treatment (Chamberlain & Hamann, 2019; Ciulli & Farnaby, 2019; Crews, 2018; Cromm & Crews, 2017; Daniels, Riching, & Urh, 2019; Deshaies, 2015; Luh et al., 2020). PROTACs result in highly specific removal of target proteins from the cell and can be used to understand protein function and cellular phenotype upon protein loss (Crews, 2018; Deshaies, 2015), providing an excellent alternative to genetic CRISPR knockout or RNA interference (RNAi) strategies (Carthew & Sontheimer, 2009; Jackson & Linsley, 2010; Pickar‐Oliver & Gersbach, 2019). PROTAC small molecules are heterobifunctional compounds that induce an interaction between a target protein with an E3 ligase component, most commonly von Hippel–Lindau (VHL) protein or cereblon (CRBN; Schapira, Calabrese, Bullock, & Crews, 2019). This induced proximity results in target protein ubiquitination and subsequent degradation via the ubiquitin proteasomal pathway (UPP; Ciulli & Farnaby, 2019; Crews, 2018; Cromm & Crews, 2017). Although numerous target‐specific PROTAC degraders have been developed, these represent only a small fraction of the total number of proteins in the proteome. Additionally, target‐specific PROTACs cannot be designed without known target binders or inhibitors that can be used as starting molecules. To overcome these challenges and provide a more general solution for targeted protein‐degradation studies, we developed the strategy of HaloPROTAC3 (Buckley et al., 2015), which degrades the 34‐kDa monomeric protein fusion tag HaloTag (Encell et al., 2012; Los et al., 2008; Urh & Rosenberg, 2012). HaloPROTAC3 comprises a VHL ligand, a linker, and a chloroalkane moiety that irreversibly binds to HaloTag (Fig. 1). The formation of a HaloTag:HaloPROTAC3:VHL ternary complex leads to ubiquitination and proteosomal degradation of the targeted HaloTag fusion protein (Fig. 1).

To understand the phenotypic consequences of protein degradation, studies that utilize endogenous proteins are critical. Not only is this essential to allow the removal of the endogenous protein, but it also ensures the preservation of the endogenous target expression level in the relevant cell backgrounds, along with target regulation from the native promoter. Recent advances in CRISPR/Cas9 have enabled genomic insertions of larger tags (Cong et al., 2013; Jinek et al., 2012; Lackner et al., 2015; Leonetti, Sekine, Kamiyama, Weissman, & Huang, 2016; Pickar‐Oliver & Gersbach, 2019; Suzuki et al., 2016), including HaloTag, although insertion efficiencies can be low, depending upon the target as well as the cell background. Fortunately, the fluorescence labeling capabilities of HaloTag (Los et al., 2008) can be leveraged at the HaloTag CRISPR pool stage to enrich edited cells via Fluorescence‐Activated Cell Sorting (FACS) sorting, even those low in target protein abundance.

For all targets of degradation compounds, detection and quantification of endogenous protein loss after treatment is challenging. Often western blot analysis is performed to monitor degradation, though this requires highly specific and sensitive target antibodies. As an alternative, we will explain the advantages of appending HiBiT (Riching et al., 2018; Schwinn et al., 2018), a short peptide tag, to HaloTag during the CRISPR insertion process to generate HiBiT‐HaloTag endogenous fusions. The HiBiT peptide, which complements with the LgBiT protein to generate NanoBiT luciferase, allows the luminescence detection of the endogenous target protein levels without the need for antibodies (Riching et al., 2018; Schwinn et al., 2018).

In this unit we present two basic protocols, the first for CRISPR/Cas9 insertion of HaloTag or HiBiT‐HaloTag, and the second for the degradation of endogenous HaloTag or HiBiT‐HaloTag fusion proteins with HaloPROTAC3. The first protocol details how to design and introduce the HaloTag and HiBiT‐HaloTag sequences onto the target protein N‐ or C‐terminal locus using CRISPR/Cas9 technology, specifically for the introduction of a larger protein fusion tag using a double‐stranded DNA (dsDNA) donor vector. After CRISPR pools are generated, further details are provided for enrichment of HaloTag positive cells using the HaloTag fluorophore, Janelia Fluor 646 (JF646) HaloTag ligand (Grimm et al., 2015) with FACS sorting. The cell‐permeable, bright JF646 HaloTag ligand, which irreversibly binds to HaloTag target fusions, has high signal to background, and therefore is highly enabling for detection and FACS of endogenous HaloTag target fusions (Chong et al., 2018; Grimm et al., 2015). The second protocol focuses on degradation experiments for endogenous HaloTag fusion proteins with HaloPROTAC3. Recommendations for HaloPROTAC3 treatments, including considerations for treatment time and concentration, are outlined. Although antibodies can be used to detect degradation, optional protocols are provided for luminescence detection of the endogenous HiBiT‐HaloTag targets and their respective CRISPR insertion efficiencies and protein levels. The coupling of these technologies allows rapid screening using luminescence to observe successful knock‐in, validate clonal selection, and/or measure degradation of the target using either endpoint lytic or kinetic live‐cell analysis.

## Basic Protocol 1 CRISPR/Cas9 INSERTION OF HaloTag OR HiBiT‐HaloTag

This protocol allows CRISPR/Cas9‐mediated genomic insertion of HaloTag or HiBiT‐HaloTag to the N or C terminus of any target protein of interest in any cell line that is amenable to CRISPR modification technology. If HiBiT is used, it should always be appended to the extreme terminus of the N‐ or C‐HaloTag insertion by including its sequence in the dsDNA CRISPR donor vector (Fig. 1). This procedure uses a purified Cas9 protein, a site‐specific guide RNA/trans‐activating CRISPR RNA (guide RNA/tracrRNA) duplex, and a dsDNA donor plasmid template containing the HaloTag or HiBiT‐HaloTag sequence flanked by 500 bp of genomic homology upstream and downstream of the insertion site. Examples are outlined with use of the Mirus Ingenio Kit and Bio‐Rad Gene Pulser Xcell Electroporation System, but the protocols can be adapted to other instruments which enable electroporation or nucleofection.

When designing the dsDNA donor plasmid, we recommend including 500 bp of homology to the target of interest. However, if this sequence is too complex for PCR or synthesis, the homology arm can be shortened to between 300 and 500 bp. If introducing HaloTag or HiBiT‐HaloTag at the N terminus of the protein, it is recommended that the tag(s) be placed immediately following the endogenous target start codons or any type of signal sequence that could be cleaved. If introducing HaloTag or HaloTag‐HiBiT at the C terminus, place the tag(s) immediately upstream of the native stop codon. Generation of genomic maps that include the CRISPR insertions will aid in ensuring the tags are placed in the proper reading frame. As CRISPR insertion success can be highly dependent upon the choice of guide RNA, it is advisable to test a minimum of two different guide RNA sequences. Initial studies appending the tag(s) on the N‐ or C‐terminal end of the protein using expression vectors can be used to determine the tag(s) effects on the protein's expression, binding interactions, and folding abilities. However, overexpression is not recommended for degradation studies with HaloPROTAC3, as little degradation will be detected due to the high expression levels of the fusion protein.

### Materials

Alt‐R CRISPR‐Cas9 tracrRNA (IDT, cat. no. 1072532)
Alt‐R CRISPR‐Cas9 CRISPR RNA (crRNA; synthesized by IDT; use IDT crRNA design tool)
Nuclease Free Duplex Buffer (IDT, cat. no. 1072570)
Alt‐R S.p. Cas9 Nuclease (IDT, cat. no. 1081058)
Desired cell line for CRISPR modification that is amenable to nucleofection
DPBS (Gibco, cat. no. 14190‐144)
0.05% trypsin/EDTA (Invitrogen, cat. no. 25300‐054) or 0.25% trypsin/EDTA (Invitrogen, cat. no 25200‐056), depending on cell line
Complete growth medium for cell type of choice
Mirus Ingenio Solution (Mirus, cat. no. MIR 50117)
HaloTag or HiBiT‐HaloTag double‐stranded (ds) DNA donor plasmid (synthesized by IDT; see recipe for sequences)
Dimethyl sulfoxide (DMSO; Sigma, cat. no. D2650‐100 ml)
Janelia Fluor 646 (JF646) HaloTag Ligand (Promega, cat. no. GA1120)
100× Antibiotic/Antimycotic solution (Gibco, cat. no. 15240‐062)
FACS buffer (see recipe)
Optional, for use of HiBiT‐HaloTag or HaloTag‐HiBiT CRISPR insertions: Nano‐GloHiBiT Lytic Detection System (Promega, cat. no. N3030)

Heat block (Thermo Scientific, cat. no. 88‐870‐001)
50‐ml conical tubes (Corning, cat. no. 352070)
Mirus Ingenio Kit (2‐mm cuvettes and bulbs; Mirus, cat. no. MIR 50118)
Bio‐Rad Gene Pulser Xcell Electroporation System
Appropriate incubator for cell line
Clear, tissue‐culture‐grade 96‐well plates (Corning, cat. no. 353072)
5‐ml round‐bottom tube with cell strainer cap (Corning, cat. no 352235)
BD FACS Melody or similar
White 96‐well plates (Corning, cat. no. 3917)
Optional, for use of HiBiT‐HaloTag or HaloTag‐HiBiT CRISPR insertions: Luminometer such as the GloMax Discover Microplate Reader (Promega, cat. no. GM3000) or CLARIOstar Plus (BMG Labtech)

Additional reagents and equipment for basic cell culture techniques, including cell counting (Phelan & May, 2017)

### Preparation of tracrRNA and crRNA RNP complexes

1 Reconstitute tracrRNA and crRNA to 100 µM using nuclease‐free duplex buffer.
  Each crRNA used will require separate duplexing and RNP complexing steps. Calculate total amounts of tracrRNA and Cas9 that will be needed for each crRNA reaction before beginning to ensure that proper amounts of each are available.
2 Prepare 50 µM tracrRNA:crRNA duplex by combining the following:

**Table:** 

Component	Volume
100 µM Alt‐R tracrRNA	20 µl
100 µM Alt‐R crRNA	20 µl
Total	40 µl
3 Heat 5 min at 95°C and allow to cool to room temperature on bench top.
4 Prepare ribonucleoprotein (RNP) complex by combining 75 pmol Cas9 and 120 pmol tracrRNA:crRNA duplex:

**Table:** 

Component	Volume
61 µM Cas9 (10 mg/ml)	1.2 µl
50 µM trRNA:crRNA	2.4 µl
Total	3.6 µl
  Add the Cas9 very slowly to avoid precipitation, and swirl with pipet tip.
5 Incubate RNP complex 10‐20 min at room temperature.
6 Determine total number of cells needed based on number of CRISPR reactions (4 × 10^6^ cells/reaction), remove cells from plasticware using appropriate splitting techniques, centrifuge cells at 200 × *g* for 5 min, rinse cells with DPBS, and centrifuge again. For example, if using HEK293 cells, wash with DPBS, detach cells with trypsin, inactivate trypsin with DMEM + 10% FBS, and transfer cells to a conical tube for centrifugation.
  The optimal density of cells used at this step can vary depending upon cell type. Refer to the Mirus Ingenio Full Electroporation Protocol for more information.

### Electroporative transfer of HaloTag or HiBiT‐HaloTag to cells

**Figure 2:**
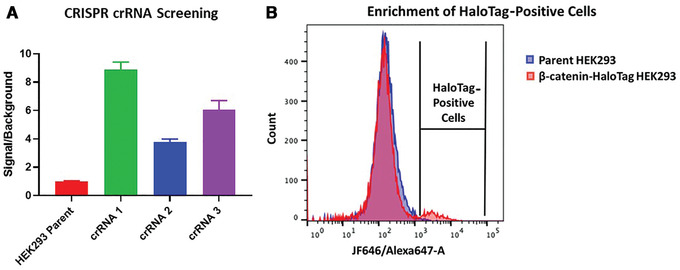
Efficiency of multiple crRNAs used to insert HaloTag‐HiBiT to the C terminus of the endogenous EPOP protein was tested using a Nano‐Glo HiBiT Lytic Detection Assay. Analyzed data from raw RLUs shows the ratio of the luminescence from each CRISPR‐edited pool of cells over background luminescence from HEK293 parent cells (**A**). crRNA 1 has the highest signal/background ratio, correlating to the highest HiBiT‐HaloTag insertion frequency. Insertion efficiency of large tags with CRISPR/Cas9 editing is low. The fluorescent JF646 HaloTag ligand allows HaloTag‐positive cells to be sorted from negative cells, enriching the edited population (**B**). Labeled parental HEK293 cells (blue) were used to determine negative cells. The β‐catenin‐HaloTag cells (red) contained a large HEK293 parent peak and a small JF646‐HaloTag‐positive peak that represented ∼6% of the total live cell population.

7 Resuspend 4 × 10^6^ cells in 100 µl per CRISPR reaction in Mirus Ingenio Solution.
8 Add 5 µg dsDNA donor plasmid to the tube containing RNP complex from step 4.
9 Add 95 µl cell suspension to the RNP/donor mixture and mix by pipetting.
10 Transfer the entire mixture to a 2‐mm electroporation cuvette from the Mirus Ingenio Kit.
  Be careful to remove any bubbles, and ensure that the cell mixture is at bottom of cuvette.
11 Insert cuvette into Bio‐Rad Gene Pulser Xcell Electroporation system and pulse cells using the recommended values for a given cell type.
  Example for HEK293 cells: 160 V, capacitance = 950, resistance = infinity, cuvette = 2.
12 Let cells recover for 5‐10 min, add 1 ml growth medium, transfer to a flask or plate, and incubate to allow recovery.
13 Allow cells to recover for 1‐2 weeks in an incubator set to the standard growth conditions (temperature/humidity/CO_2_ level) for the cell type, and split cells if they become confluent. If performing HiBiT‐HaloTag CRISPR insertions, HiBiT lytic luminescence assays can be performed to determine insertion efficiency (Fig. 2A) by following steps 27‐32.

### Obtain HaloTag CRISPR clones

To obtain a HaloTag CRISPR clone from this initial pool, FACS sorting can be performed according to the following general guidelines (though it will need to be tailored to individual FACs instruments.

14 Prepare one confluent T75 flask of CRISPR/Cas9‐edited cells and two T75 flasks of the unedited parental cell line to be used as controls.
15 Add 71.1 µl DMSO to tube of JF646 HaloTag ligand to make a 100 µM stock, then add 50 µl of this stock to 25 ml of cell culture growth medium to make a 200 nM labeling solution.
16 Remove medium from the CRISPR/Cas9‐edited cell flask and from one flask of the parental cells, and replace with 10 ml each of the 200 nM labeling solution.
17 Place cells in an incubator for 20‐30 min.
18 Reserve the second parental cell line flask in growth medium to serve as a no‐label control.
19 Prepare sort medium by adding 1× antibiotic/antimycotic to 100 ml of growth medium. For single‐cell sorting into 96‐well plates, transfer 200‐µl aliquots into each well and then place plates in incubator; it is recommended that a minimum of three 96‐well plates be used. For enrichment of HaloTag‐positive cells, transfer 1‐ml aliquots of sort medium into a 5‐ml round‐bottom tube. After sorting, cells can be transferred into tissue culture flask preincubated with the appropriate volume of sort medium.
20 Prepare FACS instrument for single‐cell sorting of cells into 96‐well plates or bulk sorting into 5‐ml round‐bottom tube.
21 Wash labeled CRISPR/Cas9‐edited and parental cells twice with DPBS. For adherent cells, trypsinize cells after the wash.
  Labeled cells can remain resuspended in full growth medium overnight to further minimize any background fluorescence of unedited cells. This may be necessary when insertion efficiencies are very low.
22 Resuspend cells in 1 ml FACS buffer.
  The amount of FACS buffer needed will depend on the cell number and rate of flow. If cells are too confluent, clogging may occur, or dispensing single‐cell clones may not be possible. If cells are too dilute, multiple wells may be left empty, and the total time spent sorting will be longer.
23 Filter cells through cell strainer cap on round‐bottom tube to remove any cell clumps. Keep cells on ice.
24 Sort cells using Alexa 647 channel for JF646 HaloTag ligand (Fig. 2B).
  - Run unlabeled parental cells to establish appropriate gating strategies to isolate live singlets by forward and side scatter.
  - Run labeled parental cells to determine background. Establish gates to collect everything with a signal intensity greater than the displayed histogram for background control cells using the Alexa 647 laser (Fig. 2B).
  - Run labeled, CRISPR/Cas9‐edited cells to sort for JF646 HaloTag ligand positive cells. Set gate above the labeled parental cell gate peak as determined in b.
    More stringent gating should yield more positive clones but potentially fewer cells, whereas less stringent gating may lead to sorting unedited cells. The histogram peak for positive cells may be small due to the low efficiency of the HiBiT‐HaloTag insertion (Fig. 2B). Even with only a small number of positive cells, multiple HaloTag‐positive cells should be recovered from the sort.
25 Incubate sort flasks or plates containing HaloTag‐positive cells in an incubator set to growing conditions for cells until cells become confluent.
26 Continue to expand cells and confirm insertion through sequencing of isolated genomic DNA.
  If cells have been sorted to have a single cell per well of a 96‐well plate, it may be several weeks before wells are confluent. Not every well containing a cell will result in formation of a colony/clonal cell line, and some cell types do not grow well from a single cell. If the insertion efficiency is very low, an initial sort of ∼10‐30 cells/well can enrich the population before single‐cell sorting.

### Optional HiBiT lytic protocol for HiBiT‐HaloTag CRISPR insertions

27 Count cells to estimate density, and resuspend to a final density of 2 × 10^5^ cells/ml in cell culture growth medium.
28 Transfer 100 µl of cells (20,000 cells) in triplicate to a white 96‐well assay plate.
29 Dilute the LgBiT Protein 1:100 and the Nano‐Glo HiBiT Lytic Substrate 1:50 into an appropriate volume of room‐temperature Nano‐Glo HiBiT Lytic Buffer in a new tube. Mix by inversion.
30 Add 100 µl Nano‐Glo HiBiT Lytic Reagent to each well.
31 Mix the samples by placing the plate on an orbital shaker (300‐600 rpm) for at least 10 min.
32 Read luminescence with GloMax Discover Microplate Reader using a 0.5‐s integration time (Fig. 2A).

## Basic Protocol 2 HaloPROTAC3 DEGRADATION OF ENDOGENOUS HaloTag FUSIONS

This protocol outlines the recommended concentrations and treatment times of HaloPROTAC3 for degradation of endogenous HaloTag or HiBiT‐HaloTag protein fusions. As the extent of degradation will be highly dependent on the endogenous expression levels of the target and its subcellular localization, ranges of concentrations and treatment times are indicated. To ensure that the observed protein loss is specific to HaloPROTAC3 and occurs through a PROTAC mechanism, performing parallel studies with the control *ent*‐HaloPROTAC3 (Buckley et al., 2015), an enantiomer of HaloPROTAC3 that is unable to engage VHL, is also recommended here.

There are several ways to detect successful degradation after HaloPROTAC3 treatment, the most common of which is use of a target‐specific antibody in a western blot application. As this will be dependent on the target as well as the antibody manufacturer, it is advisable to follow the manufacturer's guidelines for detection and antibody validation. Included in this protocol is an optional endpoint, lytic luminescence detection if HiBiT‐HaloTag CRISPR insertions are performed. Also included (steps 8‐18) is an alternate procedure for the live‐cell kinetic degradation analysis of HiBiT‐HaloTag insertions to better understand degradation rate, optimal time of treatments, and kinetic dose‐response curves. The use of HiBiT for protein level detection is highly quantitative, directly correlative to the endogenous target protein level, and does not require the use of antibodies (Riching et al., 2018; Schwinn et al., 2018; Schwinn, Steffen, Zimmerman, Wood, & Machleidt, 2020).

### Materials

HaloTag or HiBiT‐HaloTag CRIPSR/Cas9‐edited cells
DPBS (Gibco, cat. no. 14190‐144)
0.05% trypsin/EDTA (Invitrogen, cat. no. 25300‐054) or 0.25% trypsin/EDTA (Invitrogen, cat. no 25200‐056), depending on cell line
Complete growth medium for cell type of choice
HaloPROTAC3 ligand (Promega, cat. no. GA3110)
*ent*‐HaloPROTAC3 ligand (Promega, cat. no. GA4110)
Dimethyl sulfoxide (DMSO; Sigma, cat. no. 02660‐100 ml)
Optional materials for live‐cell kinetic degradation of HiBiT‐HaloTag fusions:
  LgBiT Expression Vector (Promega, cat. no. N2681)
  Nano‐Glo Endurazine Live Cell Substrate (Promega, cat. no. N2570/1/2)
  CO_2_‐independent medium (Gibco, cat. no. 18045‐088)
  FBS (VWR, cat. no. 89510‐194)

Six‐well plates
Appropriate incubator for cell line
Dilution reservoirs (Dilux, cat. no. D‐1002)
White 96‐well plates (Corning, cat. no. 3917)
Optional material for live‐cell kinetic degradation of HiBiT‐HaloTag fusions: Luminometer such as the GloMax Discover Microplate Reader (Promega, cat. no. GM3000) or CLARIOstar Plus (BMG Labtech)

### HaloPROTAC3 degradation of fusions

**Figure 3:**
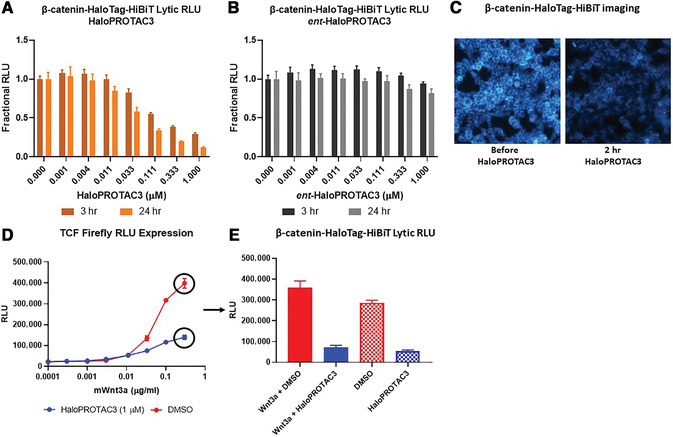
Endpoint analysis of a homozygous β‐catenin‐HaloTag‐HiBiT endogenous protein fusion in HEK293 cells that stably express LgBiT after degradation with HaloPROTAC3. Cells contained the endogenous β‐catenin‐HaloTag‐HiBiT protein fusion were treated with HaloPROTAC3 for 3 or 24 hr, and degradation was detected with an endpoint HiBiT lytic assay (**A**). HaloPROTAC3 caused rapid reduction in the amount of tagged protein in the cell, which was visualized through luminescence imaging on an Olympus LV200 microscope (**B**). Phenotypic characterization was performed on cells that were treated with HaloPROTAC3. Cells containing the endogenous β‐catenin‐HaloTag‐HiBiT protein fusion were transfected with a TCF firefly luciferase reporter before being treating with mWnt3a. HaloPROTAC3 was used to degrade the beta‐catenin‐HaloTag‐HiBiT protein fusion, and after 24 hr a lytic dual luciferase assay was performed to measure firefly luciferase expressed from the TCF reporter (**C**) and β‐catenin HiBiT luminescence (**D**). TCF expression was not induced in cells that were treated with HaloPROTAC3 because β‐catenin‐HaloTag‐HiBiT protein fusions were degraded and could not enter the nucleus upon treatment with mWnt3a.

1 Culture CRISPR/Cas9‐edited cells carrying HaloTag, HiBiT‐HaloTag (N‐terminal fusion), or HaloTag‐HiBiT (C‐terminal fusion) insertions appropriately in preparation for the assay.
  If performing live‐cell degradation assays with HiBiT‐HaloTag CRISPR insertions proceed to optional live‐cell HiBiT‐HaloTag degradation assay at the end of Basic Protocol 2.
2 For adherent cells, wash cells with DPBS and trypsinize.
3 Count cells and plate 800,000 cells per well in a six‐well plate. Plate one well for HaloPROTAC3, one for *ent*‐HaloPRTOAC3, and one for DMSO control.
4 For adherent cells, incubate cells overnight (18‐24 hr) in an appropriate incubator. For suspension cells, proceed to step 5.
5 Add ligands to growth medium in well to obtain final concentrations of (a) 300 nM HaloPROTAC3, (b) 300 nM *ent*‐HaloPROTAC3, and (c) a volume of DMSO equivalent to that added in the PROTAC treatments.
  For example, if there is 2 ml of growth medium in well, add 500 µl 1.5 µM HaloPROTAC3 stock to make a solution of 300 nM final concentration.
6 Incubate cells in an appropriate incubator overnight.
  These recommended HaloPROTAC3 concentrations and treatment times can be increased or decreased depending upon target and desired level of degradation.
7 Detect degradation of endogenous HaloTag target fusion. If using HiBiT‐HaloTag CRISPR insertions, HiBiT lytic luminescence assays can be carried out to quantitate degradation following Basic Protocol 1, steps 27‐32 (examples are shown in Figure 3A and B). To use antibodies to detect for HaloTag target protein degradation, follow recommended protocols for western blot analysis from the respective manufacturer. Alternatively, live‐cell kinetic degradation analysis of HiBiT‐HaloTag insertions can be performed to provide a more detailed understanding of the degradation process (steps 8‐18 below).

### Optional live‐cell luminescence degradation detection of HiBiT‐HaloTag CRISPR insertions

**Figure 4:**
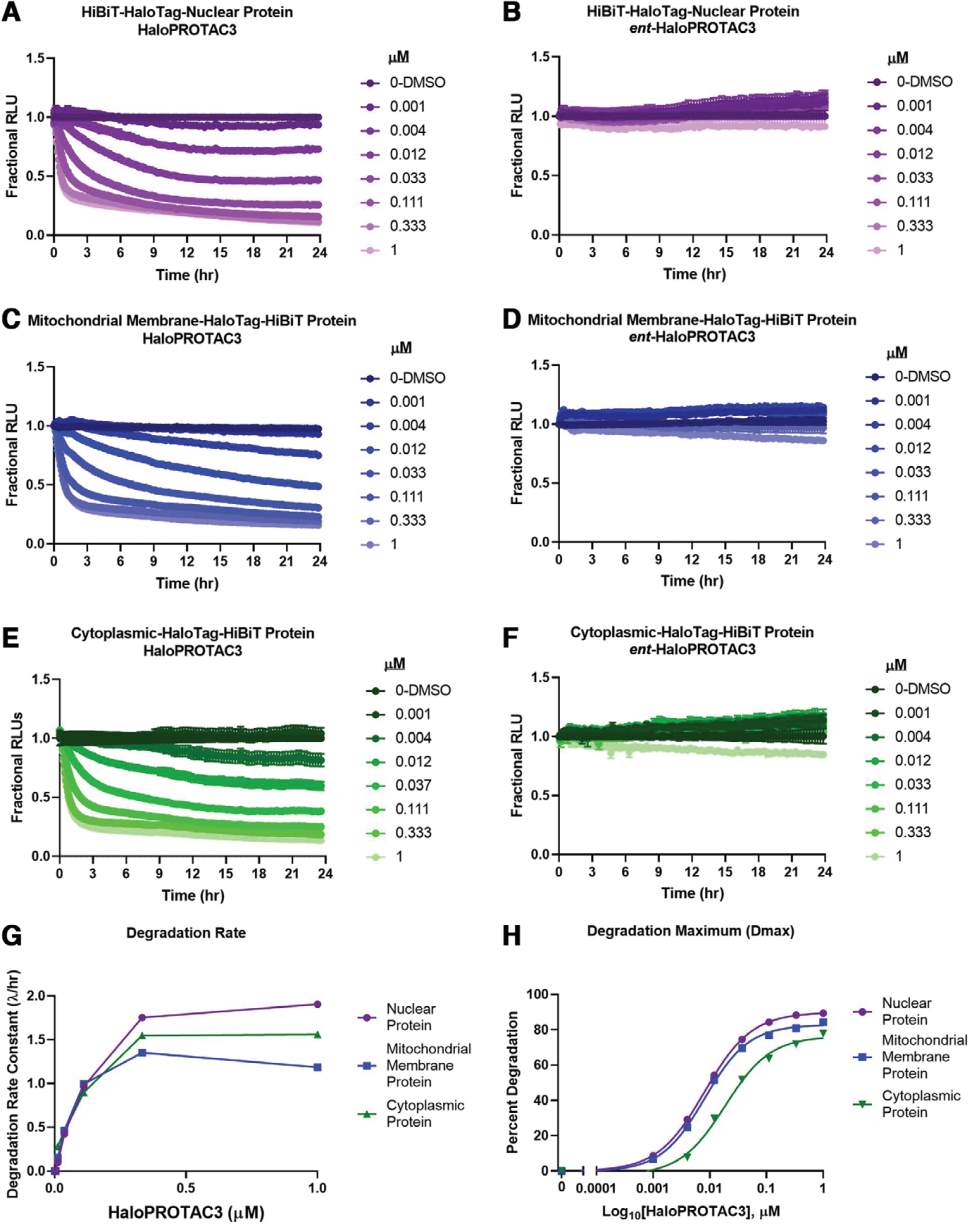
Kinetic degradation profiles of endogenous nuclear, mitochondrial membrane, and cytoplasmic HiBiT‐HaloTag protein fusions after treatment with HaloPROTAC3. Cells were treated with a series of dilutions of HaloPROTAC3 (**A**, **C**, **E**) or *ent*‐HaloPROTAC3 (**B**, **D**, **F**), and degradation was followed by luminescence detection in live cells through a kinetic degradation assay for 24 hr. An N‐terminal HiBiT‐HaloTag‐nuclear endogenous protein fusion in HEK293 cells that stably express LgBiT, a C‐terminal HaloTag‐HiBiT mitochondrial membrane endogenous protein fusion in parent HEK293 cells with LgBiT introduced by transient transfection, and a C‐terminal HaloTag‐HiBiT‐cytoplasmic protein fusion in HEK293 cells that stably express LgBiT were used, respectively. No degradation was detected in the *ent*‐HaloPROTAC3‐treated samples, confirming that the protein loss is due to a PROTAC‐mediated mechanism. (**G**) Rapid degradation rates were observed in all samples, with >50% degradation occurring in the first 3 hr. Around 80% degradation was achieved with each HiBiT‐HaloTag protein fusion regardless of cellular location (**H**). DC_50_ values were 8.1 nM for the mitochondrial membrane and nuclear protein and 18.6 nM for the cytoplasmic protein.

**Figure 5:**
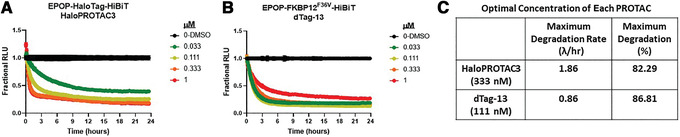
Comparison between a HaloTag insertion with HaloPROTAC3 degradation and an FKBP12^F36V^ insertion with dTag‐13 degradation. Endogenous EPOP was tagged at the C terminus with either HaloTag‐HiBiT or FKBP12^F36V^‐HiBiT in HEK293 cells. HiBiT was used in combination with both tags to allow the detection of luminescent live cells once LgBiT was introduced by transient transfection. Both HaloPROTAC3 (**A**) and dTag‐13 (**B**) produced rapid and robust degradation of the protein fusion with the ∼80% degradation after 24 hr and similar levels of compound (**C**). However, with the dTag‐13 compound (**B**), a hook effect was detected in which at higher concentrations, the rate slows and degradation decreases.

8 Transfect the LgBiT vector into HaloTag‐ or HiBiT‐HaloTag‐edited cells using standard transient transfection and following manufacturer's recommendations.
9 Incubate plates in an appropriate incubator overnight (18‐24 hr).
10 Prepare a 1× solution of Nano‐Glo Endurazine Live Cell Substrate in CO_2_‐independent medium with the appropriate percentage of FBS for the cell type, or proper assay medium for cells, by diluting the stock reagent of the substrate 1:100. If the luminometer provides CO_2_ injection or cells do not require CO_2_, regular assay medium can be used.
11 Aspirate cell culture medium from plate, and add 90 µl of the Endurazine solution to each well.
12 Incubate plate for at least 2.5 hr at in an incubator set to the appropriate growing conditions for the cells to equilibrate the luminescence. During this incubation period, it is advisable to pre‐equilibrate the luminometer to the growth temperature and CO_2_ level (if possible or needed) of cell line in use.
13 Prepare 10 µM solutions of HaloPROTAC3 and *ent*‐HaloPROTAC3 either in CO_2_‐independent medium containing the appropriate percentage of FBS for the cell type or in proper assay medium for the cells.
14 Perform threefold dilutions of 10 µM HaloPROTAC3 or *ent*‐HaloPROTAC3 in dilution reservoirs using assay medium that contains DMSO at the same concentration as the 10 µM stock. Reserve the last well of the dilution series to contain DMSO alone, as a negative control. This results in a 10× dilution series, which will be further diluted upon addition to cells for a final treatment concentration of 1 µM at the highest point.
15 Add 10 µl of the diluted HaloPROTAC3 or *ent*‐HaloPROTAC3 ligand to each well of the 96‐well plate.
16 Immediately place the plate in a luminometer plate reader pre‐equilibrated to the growth temperature of the cell line in use.
17 Read luminescence at desired intervals. A recommended starting point is every 15 min over a 24‐hr total time frame.
18 Calculate the fractional RLUs at each time point by dividing the RLU value from HaloPROTAC3‐treated or *ent*‐HaloPROTAC3‐treated wells by those from the DMSO control (fractional RLU = PROTAC‐treated RLU/DMSO RLU). Examples of kinetic degradation profiles of HiBiT‐HaloTag CRISPR insertions are shown in Figures 4 and 5.

## REAGENTS AND SOLUTIONS

### FACS buffer

1× Hank's Balanced Salt Solution (HBSS; Gibco, cat. no. 14175‐095)
10 mM HEPES (Gibco, cat. no. 15630‐080)
0.2% BSA (Millipore, cat. no. 126626‐50 ml)
1× Antibiotic/Antimycotic Solution (diluted from 100×, Gibco, cat. no. 15240‐062)

### HaloTag dsDNA donor sequences

*N‐terminal HaloTag sequence*:

500‐bp upstream homology arm‐GCAGAAATCGGTACTGGCTTTCCATTCGACCCCCATTATGTGGAAGTCCTGGGCGAGCGCATGCACTACGTCGATGTTGGT CCGCGCGATGGCACCCCTGTGCTGTTCCTGCACGGTAACCCGACCTCCT CCTACGTGTGGCGCAACATCATCCCGCATGTTGCACCGACCCATCGCTG CATTGCTCCAGACCTGATCGGTATGGGCAAATCCGACAAACCAGACCTG GGTTATTTCTTCGACGACCACGTCCGCTTCATGGATGCCTTCATCGAAG CCCTGGGTCTGGAAGAGGTCGTCCTGGTCATTCACGACTGGGGCTCCGC TCTGGGTTTCCACTGGGCCAAGCGCAATCCAGAGCGCGTCAAAGGTATT GCATTTATGGAGTTCATCCGCCCTATCCCGACCTGGGACGAATGGCCAG AATTTGCCCGCGAGACCTTCCAGGCCTTCCGCACCACCGACGTCGGCCG CAAGCTGATCATCGATCAGAACGTTTTTATCGAGGGTACGCTGCCGATG GGTGTCGTCCGCCCGCTGACTGAAGTCGAGATGGACCATTACCGCGAGC CGTTCCTGAATCCTGTTGACCGCGAGCCACTGTGGCGCTTCCCAAACGA GCTGCCAATCGCCGGTGAGCCAGCGAACATCGTCGCGCTGGTCGAAGA ATACATGGACTGGCTGCACCAGTCCCCTGTCCCGAAGCTGCTGTTCTGG GGCACCCCAGGCGTTCTGATCCCACCGGCCGAAGCCGCTCGCCTGGCCA AAAGCCTGCCTAACTGCAAGGCTGTGGACATCGGCCCGGGTCTGAATCT GCTGCAAGAAGACAACCCGGACCTGATCGGCAGCGAGATCGCGCGCTG GCTGTCGACGCTCGAGATTTCCGGCGAGCCAACCACTGAGGATCTGTAC TTTCAGAGCGATAAC‐500‐bp downstream homology arm

*C‐terminal HaloTag sequence*:

500‐bp upstream homology arm‐GAGCCAACCACTGAGGATCTGTACTTTCAG AGCGATAACGATGGATCCGAAATCGGTACTGGCTTTCCATTCGACCCCC ATTATGTGGAAGTCCTGGGCGAGCGCATGCACTACGTCGATGTTGGTC CGCGCGATGGCACCCCTGTGCTGTTCCTGCACGGTAACCCGACCTC CTCCTACGTGTGGCGCAACATCATCCCGCATGTTGCACCGACCCAT CGCTGCATTGCTCCAGACCTGATCGGTATGGGCAAATCCGACAAAC CAGACCTGGGTTATTTCTTCGACGACCACGTCCGCTTCATGGATGC CTTCATCGAAGCCCTGGGTCTGGAAGAGGTCGTCCTGGTCATTCAC GACTGGGGCTCCGCTCTGGGTTTCCACTGGGCCAAGCGCAATCCAG AGCGCGTCAAAGGTATTGCATTTATGGAGTTCATCCGCCCTATCCC GACCTGGGACGAATGGCCAGAATTTGCCCGCGAGACCTTCCAGGCC TTCCGCACCACCGACGTCGGCCGCAAGCTGATCATCGATCAGAACG TTTTTATCGAGGGTACGCTGCCGATGGGTGTCGTCCGCCCGCTGAC TGAAGTCGAGATGGACCATTACCGCGAGCCGTTCCTGAATCCTGTT GACCGCGAGCCACTGTGGCGCTTCCCAAACGAGCTGCCAATCGCCG GTGAGCCAGCGAACATCGTCGCGCTGGTCGAAGAATACATGGACTG GCTGCACCAGTCCCCTGTCCCGAAGCTGCTGTTCTGGGGCACCCCA GGCGTTCTGATCCCACCGGCCGAAGCCGCTCGCCTGGCCAAAAGCC TGCCTAACTGCAAGGCTGTGGACATCGGCCCGGGTCTGAATCTGCT GCAAGAAGACAACCCGGACCTGATCGGCAGCGAGATCGCGCGCTGG CTGTCTACTCTGGAGATTTCCGGT‐500‐bp downstream homology arm

*N‐terminal HiBiT‐HaloTag sequence*:

500‐bp upstream homology arm‐GTGAGCGGCTGGCGGCTGTTCAAGAAGA TTAGCGCAGAAATCGGTACTGGCTTTCCATTCGACCCCCATTATGTGG AAGTCCTGGGCGAGCGCATGCACTACGTCGATGTTGGTCCGCGCGATGG CACCCCTGTGCTGTTCCTGCACGGTAACCCGACCTCCTCCTACGTGTGG CGCAACATCATCCCGCATGTTGCACCGACCCATCGCTGCATTGCTCCAG ACCTGATCGGTATGGGCAAATCCGACAAACCAGACCTGGGTTATTTCTT CGACGACCACGTCCGCTTCATGGATGCCTTCATCGAAGCCCTGGGTCTG GAAGAGGTCGTCCTGGTCATTCACGACTGGGGCTCCGCTCTGGGTTTCC ACTGGGCCAAGCGCAATCCAGAGCGCGTCAAAGGTATTGCATTTATGGA GTTCATCCGCCCTATCCCGACCTGGGACGAATGGCCAGAATTTGCCCGC GAGACCTTCCAGGCCTTCCGCACCACCGACGTCGGCCGCAAGCTGATCA TCGATCAGAACGTTTTTATCGAGGGTACGCTGCCGATGGGTGTCGTCCG CCCGCTGACTGAAGTCGAGATGGACCATTACCGCGAGCCGTTCCTGAAT CCTGTTGACCGCGAGCCACTGTGGCGCTTCCCAAACGAGCTGCCAATCG CCGGTGAGCCAGCGAACATCGTCGCGCTGGTCGAAGAATACATGGACTG GCTGCACCAGTCCCCTGTCCCGAAGCTGCTGTTCTGGGGCACCCCAGGC GTTCTGATCCCACCGGCCGAAGCCGCTCGCCTGGCCAAAAGCCTGCCTA ACTGCAAGGCTGTGGACATCGGCCCGGGTCTGAATCTGCTGCAAGAAG ACAACCCGGACCTGATCGGCAGCGAGATCGCGCGCTGGCTGTCGACGCT CGAGATTTCCGGCGAGCCAACCACTGAGGATCTGTACTTTCAGAGCGAT AAC‐500‐bp downstream homology arm

*C‐terminal HaloTag‐VS‐HiBiT sequence*:

500‐bp upstream homology arm‐ GAGCCAACCACTGAGGATCTGTACTTTCAG AGCGATAACGATGGATCCGAAATCGGTACTGGCTTTCCATTCGACC CCCATTATGTGGAAGTCCTGGGCGAGCGCATGCACTACGTCGATGT TGGTCCGCGCGATGGCACCCCTGTGCTGTTCCTGCACGGTAACCCG ACCTCCTCCTACGTGTGGCGCAACATCATCCCGCATGTTGCACCGA CCCATCGCTGCATTGCTCCAGACCTGATCGGTATGGGCAAATCCGA CAAACCAGACCTGGGTTATTTCTTCGACGACCACGTCCGCTTCATG GATGCCTTCATCGAAGCCCTGGGTCTGGAAGAGGTCGTCCTGGTCA TTCACGACTGGGGCTCCGCTCTGGGTTTCCACTGGGCCAAGCGCAA TCCAGAGCGCGTCAAAGGTATTGCATTTATGGAGTTCATCCGCCCT ATCCCGACCTGGGACGAATGGCCAGAATTTGCCCGCGAGACCTTCC AGGCCTTCCGCACCACCGACGTCGGCCGCAAGCTGATCATCGATCA GAACGTTTTTATCGAGGGTACGCTGCCGATGGGTGTCGTCCGCCCG CTGACTGAAGTCGAGATGGACCATTACCGCGAGCCGTTCCTGAATC CTGTTGACCGCGAGCCACTGTGGCGCTTCCCAAACGAGCTGCCAAT CGCCGGTGAGCCAGCGAACATCGTCGCGCTGGTCGAAGAATACATG GACTGGCTGCACCAGTCCCCTGTCCCGAAGCTGCTGTTCTGGGGCA CCCCAGGCGTTCTGATCCCACCGGCCGAAGCCGCTCGCCTGGCCAA AAGCCTGCCTAACTGCAAGGCTGTGGACATCGGCCCGGGTCTGAAT CTGCTGCAAGAAGACAACCCGGACCTGATCGGCAGCGAGATCGCGC GCTGGCTGTCTACTCTGGAGATTTCCGGTGTCTCCGTGAGCGGCTG GCGGCTGTTCAAGAAGATTAGC‐500‐bp downstream homology arm

*Key*: blue, HiBiT; red, HaloTag; green, linker.

## COMMENTARY

### Background Information

Targeted protein degradation has resulted in an explosion of new avenues of research, from therapeutic drug discovery and clinical trials to the expansion of E3 ligase studies and phenotypic studies (Chamberlain & Hamann, 2019; Ciulli & Farnaby, 2019; Crews, 2018; Cromm & Crews, 2017; Deshaies, 2015; Schapira et al., 2019). Protein loss phenotypic studies using degradation compounds allow the highly precise and temporal loss of a target protein to be tailored a specific desired amount (Buckley et al., 2015; Nabet et al., 2018; Nishimura, Fukagawa, Takisawa, Kakimoto, & Kanemaki, 2009; Sathyan et al., 2019; Tovell et al., 2019). This approach is technically different from genetic CRISPR knock‐out (Pickar‐Oliver & Gersbach, 2019) or small interfering RNA (siRNA) approaches (Carthew & Sontheimer, 2009; Jackson & Linsley, 2010), which prevent the production of the protein and cannot be used to study essential proteins. To broadly enable targeted degradation studies, either for phenotype or to understand whether a protein could be degraded via the UPP, HaloPROTAC3 (Buckley et al., 2015), a small molecule degrader that irreversibly binds to and degrades HaloTag along with its fusion partners in live cells, was developed (Fig. 1). HaloPROTAC3 elicits degradation through formation of a ternary complex with a target HaloTag fusion protein and VHL, an E3 ligase component (Fig. 1). This results in ubiquitination of HaloTag and HaloTag target fusions and subsequent degradation by the proteasome (Fig. 1).

Several targeted protein degradation methods that utilize fusion tags or short degron epitopes have been developed (Buckley et al., 2015; Nabet et al., 2018; Nishimura et al., 2009; Sathyan et al., 2019; Tovell et al., 2019). These systems overcome the challenges involved in the design and development of successful target‐specific degradation compounds (Crews, 2018) and provide broad and robust applicability for numerous targets. The chosen target for fusion‐tag degradation studies must be accessible for recruitment into the UPP for degradation and expressed as a fusion tag protein (Buckley et al., 2015; Nabet et al., 2018; Nishimura et al., 2009; Sathyan et al., 2019; Tovell et al., 2019). In addition to HaloTag and HaloPROTAC3, discussed above, other systems include the auxin‐inducible degron (AID; Nishimura et al., 2009; Sathyan et al., 2019) and the FKBP^F36V^ dTAG (Nabet et al., 2018) systems. The AID system utilizes a degron tag appended to the target protein yet requires exogenous expression of a non‐native plant F‐box protein as well as addition of auxin to induce degradation (Nishimura et al., 2009). Although this has been shown to successfully degrade several proteins, it has proved to be challenging for in vivo models. The dTAG system (Nabet et al., 2018) conceptually is more similar to HaloPROTAC3. Target proteins are expressed as FKBP^F36V^ fusion proteins and recruited into a ternary complex with dTAG PROTAC and CRBN. A challenge with the dTAG system is the limited functionality of the FKBP^F36V^ protein, which cannot be labeled fluorescently to enrich for CRISPR insertion or be used to further understand protein function. Both dTAG and HaloPROTAC3 work well for in vivo studies (BasuRay, Wang, Smagris, Cohen, & Hobbs, 2019; Nabet et al., 2018). In mice, target HaloTag fusions have been introduced genetically or via xenographs into the organism and then efficiently degraded through HaloPROTAC3 injection (BasuRay et al., 2019). The HaloTag fusions can additionally be fluorescently labeled to study protein localization as well as isolated using a HaloTag resin to study protein interactions (Urh & Rosenberg, 2012).

### Critical Parameters and Troubleshooting

#### Basic Protocol 1: CRISPR/Cas9 insertion of HaloTag or HiBiT‐HaloTag

The use of CRISPR/Cas9 application for insertion of sequences >500 bp requires use of dsDNA donor vectors and longer homology arms (300‐500 bp each) as compared to smaller insertions, for which single‐stranded oligodeoxynucleotide (ssODN) synthesis is possible and homology arms (30‐50bp each) are shorter. The use of dsDNA donor vectors in general results in low insertion efficiency, and expected pool percentage of edited cells with this approach could range between 0.1% and 15%. For insertion of HaloTag or HiBiT‐HaloTag, the use of donor vector without a promoter is important to promote specific, on‐target insertion and minimize random integration of vector, which could then result in expression of the tag alone. To identify CRISPR HaloTag target‐edited cells from random integration, it is important to assess that the proper‐sized fusion is made by amplifying the genomic region by PCR or visualizing protein size on a protein gel with the HaloTag TMR ligand (Los et al., 2008). Sequencing is also necessary to assess zygosity and ensure the proper genomic insertion. The fluorescent HaloTag ligands used for FACS enrichment can aid these protein functional characterizations as well by assessing proper localization in the cell by confocal microscopy (Los et al., 2008).

To further optimize the success of a HaloTag CRISPR insertion, it is advisable to test multiple guide RNAs and to choose a cell line that has high electroporation or nucleofection efficiency, if possible. If inserting the HiBiT‐HaloTag sequence, the resultant pools from varying guides or electroporation settings can readily be screened for luminescence to see which conditions are most favorable for further optimization. If there is no preference for choice in termini, oftentimes insertion of tags at the extreme C terminus is easier in design, though N‐terminal insertion can also be done. With N‐terminal insertions it is important to place the tag directly following the starting ATG codon of the target protein, whereas with C‐terminal insertions, the tag should be placed immediately preceding the endogenous stop codon. Even with insertion efficiency as low as 1%, the ability to fluorescently label HaloTag positive cells with JF646 HaloTag ligand coupled with FACS enables a path to enriched pool populations or single‐cell clones that would otherwise be very challenging to achieve with blind sorting or limiting dilution approaches.

Ideally, for HaloPROTAC3 phenotypic studies, HaloPROTAC3 should degrade all copies of the target protein in the cell. For this reason, homozygous allelic HaloTag insertion is desired. However, due to the low efficiency, homozygous allelic insertions are rare as compared to heterozygous insertions, and many clones need to be screened to identify a homozygous insertion. Droplet digital PCR can be applied after FACS to identify heterozygous versus homozygous insertions before Sanger sequencing for final confirmation. Alternatively, heterozygous clones, particularly those with the insertion targeted to the N terminus of the protein, often contain small insertions or deletions (INDELs) on untagged alleles, resulting in a knockout of the untagged protein copies. As a result, the entire target protein pool in these clones is expressed as a fusion to HaloTag; therefore, these clones are sufficient for phenotypic experiments with HaloPROTAC3.

#### Basic Protocol 2: HaloPROTAC3 degradation of endogenous HaloTag fusions

HaloPROTAC3 will degrade HaloTag target fusion proteins that are recruited to the VHL E3 ligase component, incorporated into active E2/E3 ligase complexes for ubiquitination, and then trafficked to the proteasome. VHL is expressed throughout the cytoplasm and nucleus, as well as in numerous cell types (Buckley et al., 2012). When performing studies with HaloPROTAC3, it is important to be certain the cell type used expresses VHL (Buckley et al., 2012), and the HaloTag on the fusion protein is presented to the cytoplasm or nucleus. HaloTag itself contains numerous lysine residues (Encell et al., 2012) that can be ubiquitinated; therefore, it is not necessary that the target protein contain lysines available for ubiquitination. Single‐pass transmembrane (TM) proteins are degraded with PROTAC compounds (Burslem et al., 2018; Huang et al., 2018), and recently the first example of a multipass mitochondrial TM protein was shown as well (Bensimon et al., 2020). It is possible that certain HaloTag target fusion proteins are in higher‐order complexes, and these structures may prevent engagement with VHL or incorporation into the productive ubiquitination complexes required to drive degradation of the HaloTag fusion. In these cases, changing the terminus where HaloTag is located may help, but there may be some situations where structural incompatibility may result in ineffective target degradation.

As endogenous target proteins have highly variable expression levels that will not be significantly altered by HaloTag insertion, varying concentrations of HaloPROTAC3 may be necessary for successful and maximal degradation. Because HaloPROTAC3 binds irreversibly to HaloTag (Buckley et al., 2015), it is not likely to act catalytically; therefore, the HaloPROTAC3 concentration needed is directly proportional to the amount of target protein. The recommended initial concentration to test is 300 nM, but this could vary from low nanomolar to low micromolar depending upon the target. Dose‐response series and different time points or kinetic degradation runs can be performed to determine the optimal dose of HaloPROTAC3 for the HaloTag fusion. In vivo studies would require multiple routes, doses, and time to be tested before an optimal HaloPRTOAC3 concentration could be chosen for downstream applications. As HaloPROTAC3 is nontoxic at these concentrations, cell viability is not impacted. The other parameter for optimization of HaloPROTAC3 degradation is time. Among the endogenous HaloTag fusions tested, all showed rapid and sustained degradation. Depending on the goals of any particular degradation study, the desired amount of degradation can be regulated by both the concentration and the duration of treatment, and these parameters can be well defined by monitoring the kinetics of degradation via luminescence with HiBiT. It is also important to note that if the HaloTag CRISPR insertion is heterozygous, treatment with the HaloPROTAC3 compound will degrade only the tagged endogenous protein. If full target knockout is required for phenotypic studies, homozygous CRISPR insertion or a heterozygous insertion with an INDEL in the untagged copy will be necessary.

With any PROTAC degradation study, it is important to be certain that observed target protein loss is due to the specific PROTAC mechanism. For endogenous HaloTag proteins, this can be achieved using *ent*‐HaloPROTAC3 (Buckley et al., 2015). *ent*‐HaloPROTAC3 has significantly reduced affinity for VHL engagement (Buckley et al., 2015), but this low affinity can be overcome with very high concentrations; therefore, it is not recommended for use as a negative control beyond 1 µM. It is also possible that degradation of the target protein with HaloPROTAC3 may induce cell death, as may be the case for essential endogenous HaloTag target fusions. If so, orthogonal cell viability assays, such as CellTiter Glo (Promega, cat. no. G7570) or CellTox Green (Promega, cat. no. G8741), will be important to deconvolute protein loss from cell death. In addition, as these are live‐cell assays, it is recommended that the DMSO concentration after addition of HaloPROTAC3 be maintained below 0.5% by volume after addition to the cells.

For the alternate procedures utilizing HiBiT‐HaloTag fusion proteins, protein levels are easily measured with luminescence (Daniels et al., 2019; Riching et al., 2018; Schwinn et al., 2018). If low or no luminescence results with HiBiT lytic assays, it is critical to confirm by sequencing and blotting techniques that the cells carry the HiBiT‐HaloTag CRISPR insertion with 100% sequence conformity and express the full protein fusion. If low or no luminescence is measured in the live‐cell assay, be certain that the cells are expressing the LgBiT protein. Performing a HiBiT lytic endpoint assay (Nano‐Glo HiBiT Lytic Detection System, Promega, cat. no. N3030) will determine whether LgBiT is present. Purified LgBiT protein could be added to ensure the HiBiT and luminescence substrate are functional. This can be separately confirmed with parental cells expressing the LgBiT vectors and then addition of a purified HiBiT protein as a control.

### Understanding Results

In the schematic shown in Figure 1, the first step towards the degradation of endogenously tagged HaloTag fusions is the introduction of HaloTag or HiBiT‐HaloTag into the target genomic locus through CRISPR/Cas9. In Basic Protocol 1, it is advisable to test several guide RNAs during the initial electroporation step to increase the chances of successful insertion at a chosen target terminus. Shown in Figure 2A are signal‐to‐background (S/B) ratio differences due to varying insertion efficiencies of multiple crRNAs chosen for tagging of the N terminus of elongin BC and Polycomb repressive complex 2–associated protein (EPOP) with HiBiT‐HaloTag in HEK293 cells with the same dsDNA vector. The crRNAs have distinct sequences, which will guide Cas9 to cut at various locations around the desired insertion site on the genome. A HiBiT lytic luminescence assay was performed on the resultant CRISPR pools and the un‐edited parental cell line. The S/B ratio was determined and the results illustrate how the choice of crRNA can significantly influence the efficiency of insertion at a chosen target site (Fig. 2A). CRISPR pools can then be further enriched for HaloTag‐positive cells using live‐cell fluorescence labeling of HaloTag with the JF646 ligand followed by FACS. Shown in Figure 2B is an example of an expected FACS histogram overlay of unedited parental HEK293 cells and CRISPR pools of HEK293 β‐catenin‐HaloTag labeled with JF646. As expected, due to the low efficiency of CRISPR insertion of HaloTag within a CRISPR pool (<5% edited cells in the total cell population), many of the cells in the HEK293 β‐catenin‐HaloTag CRISPR pools are unedited, overlapping with the parental HEK293 cell line (Fig. 2B). However, the HaloTag‐positive cells, though only a small fraction of the total population, could easily be identified and separated from the unedited population, allowing the enrichment of those with the HaloTag insertion (Fig. 2B). This approach can be used to generate enriched mini‐pools of HaloTag‐positive edited cells or single‐cell clones even in cases in which only 1% of the cell population contains the HaloTag insert.

As an example of HaloPROTAC3 degradation and phenotype studies of a target with no available degraders or specific PROTACs, a homozygous CRISPR clone of β‐catenin‐HaloTag‐HiBiT was generated in HEK293 cells stably expressing LgBiT. In Figure 3A, we found that a HaloPROTAC3 concentration of 333 nM degraded ∼60% of the β‐catenin by 3 hr and ∼80% by 24 hr using the optional HiBiT lytic detection protocol outline in Basic Protocol 2 (Fig. 3A). Degradation was not observed with *ent*‐HaloPROTAC3 at either 3 or 24 hr (Fig. 3B), demonstrating that the protein loss is by the PROTAC‐mediated mechanism. As HiBiT can also be used for imaging upon live‐cell complementation with the expressed LgBiT protein, luminescence imaging was performed before and after 1 µM HaloPROTAC3 treatment for 2 hr (Fig. 3C). These results showed that the endogenous β‐catenin‐HaloTag‐HiBiT is properly localized at the plasma membrane, cytoplasm, and nucleus (Clevers & Nusse, 2012; Nusse, 2012) and degradation appears to occur of all populations of β‐catenin‐HaloTag‐HiBiT (Fig. 3C). Because the HaloTag‐HiBiT tag is added to β‐catenin upon synthesis, all populations of the protein will be labeled and therefore degraded by HaloPROTAC3, which binds all available HaloTag regardless of location. To study β‐catenin phenotypic response to Wnt signaling and activation of the canonical Wnt pathway (Clevers & Nusse, 2012; Nusse, 2012), an orthogonal firefly Tcf reporter was transfected into the CRISPR β‐catenin‐HaloTag‐HiBiT HEK293 cells (Figs. 3D and E). Upon Wnt3a treatment, β‐catenin accumulates and enters the nucleus, binds to and initiates transcription of TCF/Lef genes (Clevers & Nusse, 2012; Nusse, 2012), including the firefly TCF luminescent reporter (Fig. 3D and E). Treatment of Wnt3a in the presence of a constant 1 µM concentration of HaloPROTAC3 results in a muted response of Tcf reporter activation (Fig. 3D) and loss of β‐catenin even in the presence of Wnt3a stimulation (Fig. 3E).

To demonstrate the ability to degrade endogenous HaloTag proteins at a variety of cellular locations, as well as to understand the quantitative parameters of HaloPROTAC3 degradation, the HiBiT kinetic studies outlined in the optional live‐cell luminescence degradation detection of HiBiT‐HaloTag CRISPR insertion protocol steps were performed (Fig. 4). For these studies, three different HaloTag‐HiBiT CRISPR clones were generated to different targets at different locations: nuclear (Fig. 4A and B), mitochondrial membrane (Fig. 4C and D), and cytoplasmic (Fig. 4E and F). The nuclear and cytoplasmic fusions were created in HEK293 cells that stably express LgBiT, and the mitochondrial membrane fusion was transfected with the LgBiT vector; all cells were then treated with an 8‐point dilution series of HaloPROTAC3 or the negative control *ent*‐HaloPROTAC3 (Fig. 4A‐F) to produce a dose‐response curve. Luminescence was continuously monitored for 24 hr, and fractional RLU, normalized to the DMSO control, was calculated to generate full degradation profiles (Fig. 4A‐F). All three target proteins from the different cellular compartments showed degradation by HaloPROTAC3 (Fig. 4A, C, and E), including the single‐pass mitochondrial membrane protein (Fig. 4C). This is now the second example of a transmembrane mitochondrial protein showing degradation (Bensimon et al., 2020), suggesting that this class of membrane proteins are amenable to degradation via PROTACs. The lack of degradation with the *ent*‐HaloPROTAC3 shows that protein loss of each of these targets is specific to HaloPROTAC3. Each of the HaloPROTAC3 degradation profiles was then used to calculate the degradation rate (Fig. 4G), as well as the degradation maximum (Dmax) and Dmax_50_, the concentration of HaloPROTAC3 that gave half the degradation maximum (Fig. 4H), for all three endogenous HiBiT‐HaloTag targets. The degradation rate is calculated by fitting a single‐component exponential decay model to each curve until the data reaches a plateau (Riching et al., 2018). The similarity of the rates and Dmax_50_ values for each of these targets indicates that it is primarily HaloTag which is driving the degradation, with minimal influence by the different targets. This is desirable for a fusion tag PROTAC system as it needs to be broadly applicable to numerous targets. The kinetic analysis also shows how the optimal dose of HaloPROTAC3 and time of treatment to achieve degradation can be clearly understood from the profiles, saving significant time and yielding more detailed information as compared to western blot analysis.

To quantitatively compare HaloPROTAC3 degradation with the dTAG PROTAC system, CRISPR clones of the EPOP target protein were generated by insertion of either HaloTag‐HiBiT or FKBP12^F36V^‐HiBiT at the C terminus in HEK293 cells (Fig. 5). These cells were transfected with the LgBiT vector to enable HiBiT kinetic degradation detection, and dose‐response curves for HaloPROTAC3 (Fig. 5A) or dTAG‐13 (Fig. 5B) were obtained. The resultant degradation profiles showed that both HaloPROTAC3, which recruits VHL (Buckley et al., 2015), and dTAG‐13, which recruits CRBN (Nabet et al., 2018), showed rapid and robust degradation of the endogenous EPOP target. The dTAG‐13 showed a hook effect (whereby the rate slows and degradation decreases at higher concentrations) at concentrations >100 nM (Fig. 5B), which was not observed with HaloPROTAC3 (Fig. 5A). A hook effect can occur when unfavorable binary complexes can form due to the high amount of PROTAC present or affinity differences between the E3 ligase and target binding ligands (Ciulli & Farnaby, 2019). This results in a slowing of the degradation rate, as observed with dTag‐13 (Fig. 5B). The HaloPROTAC3 degraded the endogenous target at a faster initial rate, and both showed nearly identical Dmax at the optimal concentrations for each PROTAC (Fig. 5C). Together, we conclude these approaches are highly similar in ability to degrade their targets yet differ significantly in ease of CRISPR editing, as the FKBP12^F36V^ has low efficiency of insertion and edited cells cannot easily be identified or fluorescently enriched from the CRISPR pool.

### Time Considerations

Basic Protocol 1: Depending upon the target, the time required for computational design of guide RNAs and donor DNAs, including homology arms, is ∼1‐2 hr. Several commercial programs exist to aid with this development. Including preparation of cells, the initial CRISPR electroporation requires 2‐3 hr of laboratory time. The cell recovery from the electroporation varies greatly depending on the cell type and its growth rate, but this commonly requires 2‐3 weeks of passaging and incubation. Sorting of the cells via FACS requires 3‐4 hr, dependent on the instrumentation (how fast the instrument can sort out positive cells) as well as the concentration of the cells. Cell recovery post‐FACS again is highly dependent upon cell type and growth rates; therefore, this can vary between 2 and 8 weeks. This length of time will be shorter as higher numbers of cells are sorted into each well (mini‐pools) as compared to single cell clones.

Basic Protocol 2: HaloPROTAC3 degradation is rapid and robust, and the time required depends on the target, as some are degraded to near completion within 5‐6 hr, and other require 24‐30 hr of treatment. Day 1 involves plating the cells for the assay. On day 2, the cells are treated, which can take up to 1 hr. They are then incubated for the time needed for degradation (typically 6‐30 hr) and then protein levels are assessed. If performing a HiBiT lytic assay to determine endogenous protein levels, this takes ∼1 hr. If using antibodies and western blot analysis, typically this requires 1‐2 days and a target‐specific antibody.

Optional HiBiT Lytic and kinetic protocols: The HiBiT lytic assay to determine insertion efficiency within CRISPR pools or measure degradation of HiBiT‐HaloTag target proteins takes 30‐60 min. The kinetic degradation assay takes 3 days to perform including plating, Endurazine equilibration, HaloPROTAC3 degradation, and data analysis.

### Author Contributions

**Elizabeth A. Caine**: Data curation; formal analysis; investigation; writing‐original draft; writing‐review & editing. **Sarah D. Mahan**: Data curation; methodology. **Rebecca L. Johnson**: Methodology. **Amanda N. Nieman**: Methodology. **Ngan Lam**: Methodology; supervision. **Curtis R. Warren**: Conceptualization; supervision. **Kristin M. Riching**: Conceptualization; formal analysis; investigation. **Marjeta Urh**: Conceptualization; supervision; writing‐original draft; writing‐review & editing. **Danette L. Daniels**: Conceptualization; data curation; formal analysis; investigation; project administration.
